# Conserving wildlife in a changing world: Understanding capture myopathy—a malignant outcome of stress during capture and translocation

**DOI:** 10.1093/conphys/coz027

**Published:** 2019-07-05

**Authors:** Dorothy Breed, Leith C R Meyer, Johan C A Steyl, Amelia Goddard, Richard Burroughs, Tertius A Kohn

**Affiliations:** 1 Division of Exercise Science and Sports Medicine, Department of Human Biology, University of Cape Town, Cape Town, South Africa; 2 Department of Paraclinical Sciences, University of Pretoria, Onderstepoort, South Africa; 3 Department of Companion Animal Clinical Studies, University of Pretoria, Onderstepoort, South Africa; 4 Department of Production Animal Studies, University of Pretoria, Onderstepoort, South Africa; 5 Centre for Veterinary Wildlife Studies, University of Pretoria, Onderstepoort, South Africa; 6 Mammal Research Institute, University of Pretoria, Onderstepoort, South Africa; 7 Biodiversity Management Branch, Environmental Management Department, City of Cape Town, Maitland, South Africa

**Keywords:** Capture stress, exertional heatstroke, hyperthermia, malignant hyperthermia, myoglobinuria, myopathy

## Abstract

The number of species that merit conservation interventions is increasing daily with ongoing habitat destruction, increased fragmentation and loss of population connectivity. Desertification and climate change reduce suitable conservation areas. Physiological stress is an inevitable part of the capture and translocation process of wild animals. Globally, capture myopathy—a malignant outcome of stress during capture operations—accounts for the highest number of deaths associated with wildlife translocation. These deaths may not only have considerable impacts on conservation efforts but also have direct and indirect financial implications. Such deaths usually are indicative of how well animal welfare was considered and addressed during a translocation exercise. Importantly, devastating consequences on the continued existence of threatened and endangered species succumbing to this known risk during capture and movement may result. Since first recorded in 1964 in Kenya, many cases of capture myopathy have been described, but the exact causes, pathophysiological mechanisms and treatment for this condition remain to be adequately studied and fully elucidated. Capture myopathy is a condition with marked morbidity and mortality that occur predominantly in wild animals around the globe. It arises from inflicted stress and physical exertion that would typically occur with prolonged or short intense pursuit, capture, restraint or transportation of wild animals. The condition carries a grave prognosis, and despite intensive extended and largely non-specific supportive treatment, the success rate is poor. Although not as common as in wildlife, domestic animals and humans are also affected by conditions with similar pathophysiology. This review aims to highlight the current state of knowledge related to the clinical and pathophysiological presentation, potential treatments, preventative measures and, importantly, the hypothetical causes and proposed pathomechanisms by comparing conditions found in domestic animals and humans. Future comparative strategies and research directions are proposed to help better understand the pathophysiology of capture myopathy.

## Teaser

The rapid threat of extinction confronts mammals globally and potentially affects almost a quarter of mammals on the African continent. The threats facing wildlife are numerous and include climate change, habit loss and poaching. The need to capture and translocate wildlife is essential to attempt to conserve species and maintain ecological balance. Stress during these operations is inevitable, and a life-threatening condition called *capture myopathy* is still the cause for most deaths during and after such efforts. There is an urgent need to understand and address the cause(s) for this condition to improve conservation efforts and enhance animal welfare during such operations.

## Introduction

Globally over the past 40 years 25% of carnivores and ungulates have moved towards extinction (Cardillo *et al.*, 2005; Di Marco *et al.*, 2014). Within African protected areas, large mammal populations have reduced by 59% in the same time period, and currently 23% of (African) mammals are threatened according to the International Union for Conservation of Nature's Red List of Threatened Species (IUCN) Red List (Schipper *et al.*, 2008; Di Marco *et al.*, 2014).

Introducing or reintroducing species through translocation to areas in their former range is widely and increasingly practiced in response to aggravating loss of habitat, devastating poaching rates and the hotter and drier climates experienced (Tarszisz *et al.*, 2014; Brown, 2015). Climate change in continents that are already largely dry such as Africa has already resulted in decreased habitable areas for wildlife (Brown, 2015). There are more translocation efforts with the aim of restoring ecological balance or preserving individual threatened species as more species require conservation efforts (Tarszisz *et al.*, 2014). The introduction of extralimital large mammal species is also widely practiced for economic and public game viewing purposes in protected areas and often requires intensive active management (including movement of animals) thereafter (Miller *et al.*, 2013; Bro-Jorgensen and Mallon, 2016).

Translocation of wildlife is a widely used conservation tool but unfortunately can have a low success rate (Dickens *et al.*, 2010; Tarszisz *et al.*, 2014). The reasons for this have not been clearly identified—it may be related to the lack of proper investigation of deaths and long-term follow up of translocated individuals and groups (Dickens *et al.*, 2010).

The physiological mechanisms that ensure the survival of a life form when its homeostasis is altered (such as fleeing from a predator, reproduction or exercise) are inherent to that organism. Capture myopathy is a pathophysiological manifestation where the inherent biological stress defences of an animal have failed or are in the process of failing (Dickens *et al.*, 2010; Blumstein *et al.*, 2015). It is a condition that causes marked morbidity and mortality predominantly in wild animals from around the world (Spraker, 1993; La Grange *et al.*, 2010; Paterson, 2014) and is often documented during capture and translocation procedures in Southern Africa and Africa (Garai *et al.*, 2005; Grange and La Grange, 2006; Oberem and Oberem, 2011; Atkinson *et al.*, 2012). It is not limited to land mammals, but the literature has reported sea mammals, reptiles and birds that acquired and succumbed to this fatal condition (Rogers *et al.*, 2004; Ward *et al.*, 2011; Phillips *et al.*, 2015; Sierra *et al.*, 2017).

The animal welfare issues related to the management and translocation of wildlife can be highly emotive. There is an increased awareness of the plight of wildlife around the globe and the need for their ethical treatment. The tragic high mortality rate of black rhinoceroses losses during the much publicized translocation projects in Kenya and South Africa in 2018 received severe criticism, due to what was perceived as preventable deaths (Africa Geographic, 2018; News24, 2018). Increasingly, where the intention has been a major conservation objective, losses cannot be afforded.

Although the well-being of animals is considered in translocations by conservators, in-depth knowledge of the physiology of individuals and species is still largely lacking (Tarszisz *et al.*, 2014). The rate of capture myopathy during and after translocation, as a malignant outcome of stress, can be indicative of how well animal welfare in a species was addressed in a capture operation (McKenzie, 1993; Dechen Quinn *et al.*, 2014). Knowledge of a species, handling and expertise all assists in reducing stress during translocation (Dechen Quinn *et al.*, 2014).

Conventionally, it is believed to arise from the inflicted stress and physical exertion that typically occur with prolonged or short intense pursuit, capture, restraint or transportation of wild animals (Wallace *et al.*, 1987; Hartup *et al.*, 1999). The condition is not only limited to animals of the wild subjected to capture (and translocation) but may also occur in zoo animals, as they may be chronically stressed due to confinement (Dickens *et al.*, 2010; Mason, 2010). Clinically, the animal usually presents with a combination of any of the following signs: lethargy, muscular stiffness, weakness, incoordination, recumbence, partial paralyses (paresis), metabolic acidosis, myoglobinuria and death (Chalmers and Barrett, 1977; Spraker, 1993). Macropathology typically reveals muscle necrosis, dark red-stained renal medullae and dark-coloured urine (Harthoorn and Van der Walt, 1974). Variations on these classic macroscopic findings exist and seem dependent on various factors.

There is no specific duration before death sets in, but death can occur within a few minutes, hours, days or even weeks after the precipitating event (Harthoorn, 1976; Spraker, 1993). The condition carries a grave prognosis, but there are occasional reports of successful treatment (Spraker, 1993; Rogers *et al.*, 2004). Intensive efforts are needed for treatment, and these are normally prolonged, mostly non-specific and supportive in nature (Rogers *et al.*, 2004; Smith *et al.*, 2005; Businga *et al.*, 2007). Therefore, in the wild, treatment is often not feasible (Paterson, 2014).

This review provides a broad overview of what is known about capture myopathy in wildlife. The focus will be on the clinical and pathophysiological presentation of the condition, potential treatments, preventative measures and, importantly, the proposed biological causes and hypothesized mechanisms. In order to improve our understanding and to devise new strategies to study this condition, capture myopathy will be compared to other myopathy-causing conditions that present with similar pathophysiology in humans and domestic animal species.

## Background of capture myopathy

The first recorded pathological description of capture myopathy was from 1964 in a Hunter’s hartebeest (*Beatragus hunteri*), currently one of the most critically endangered antelope species (Jarrett *et al.*, 1964). Since this first recording, more veterinarians and conservationists started recognizing the condition and reported it in the literature. However, very little research has been performed to better understand the pathogenesis or biology thereof. The condition also goes by many names, including ‘capture stress’ or ‘white muscle disease’, ‘exertional rhabdomyolysis’, ‘transit myopathy’, ‘diffuse muscular degeneration’, ‘stress myopathy’, ‘muscular dystrophy’ and ‘idiopathic muscle necrosis’, but ‘capture myopathy’ is the preferred term (Jarrett *et al.*, 1964; Spraker, 1993). As a result of its similar presentation, capture myopathy has been compared to a human condition known as either exertional rhabdomyolysis or exertional heatstroke.

Capture and translocation of wildlife are essential tools in wildlife conservation and management, and both have played a major role in securing the survival of many species. In Southern Africa, capture and relocation of wildlife have become commonplace due to the multibillion-dollar private game farming industry that has developed over the past four decades (Endangered Wildlife Trust, 2016). Largely as a result of this industry, the conservation and economic value of certain species have increased dramatically, with many individual wild animals reaching auction prices exceeding a million US dollars (Bosveld Vleissentraal, 2017). Thus, the loss of an individual animal may present a conservation or substantial economic loss. In addition, more wildlife has succumbed to capture myopathy than any other disease in the past few decades (La Grange *et al.*, 2010). The high financial risk to the loss of wild animals from capture myopathy thus emphasizes the importance to better understand the causes, treatment and prevention of this fatal condition.

Human presence, restraint and the added fear of motorized vehicle noise during capture have been hypothesized to be the primary stressors that activate the ‘fight or flight’ response in these animals. Spraker (1993) proposed that good management practises reduce the stress that the animals experience during capture and relocation, lowering the incidence of capture myopathy to <2%. Despite these recommendations, capture myopathy still occurs, primarily from not knowing the underlying pathophysiological mechanisms causing this condition and how stress and exertion can give rise to muscle damage (La Grange *et al.*, 2010; Mason, 2010).

Finally, there are no published data accurately reflecting how many animals die from capture myopathy. The reasons may vary but could merely be kept secret for financial or animal welfare reasons or just not recorded at all and perceived as unimportant.

## Capture myopathy presentation in animals

### Pathophysiology

The condition of capture myopathy is an often fatal, exertion- or stress-induced muscle degenerative condition affecting captured wild animals. The myopathy referred to relates to the muscle damage and weakness observed after a strenuous event. Muscle damage (rhabdomyolysis) is central to the pathogenesis of capture myopathy (Harthoorn, 1976; Spraker, 1993).

When the basal membrane and sarcolemma of the injured muscle fibres are compromised (such as in rhabdomyolysis), the consequential result is that cytoplasmic components, such as myoglobin and creatine kinase (CK), are released from the injured muscle fibres into the blood stream (Spraker, 1993; Vanholder *et al.*, 2000). In addition, blood lactate concentration is elevated leading to a decrease in pH and acidosis. Although not confirmed, this change in metabolism may contribute to the high body temperatures observed in the early stages of capture myopathy (Meyer *et al.*, 2008; La Grange *et al.*, 2010).

Acute kidney injury often occurs and is believed to be one of the devastating effects associated with myoglobinaemia. Myoglobinuric acute kidney injury is induced by three mechanisms: (i) vasoconstriction, (ii) intraluminal cast formation and (iii) haem–protein-induced cytotoxicity (Vanholder *et al.*, 2000). Prolonged splanchnic vasoconstriction from the fight or flight phase of the stress response may also result in renal ischaemia. The vasoconstriction, causing decreased renal perfusion, results in hypoxic damage to the glomeruli and tubules and proteinuria that can cause the obstruction of the renal tubules, reducing the glomerular filtration rate (Spraker, 1993; Vanholder *et al.*, 2000). Myoglobin is usually easily filtered by the glomerular basement membrane, but the underlying metabolic acidosis is a driver for myoglobin precipitation, which results in cast formation. A breakdown product from myoglobin, called ferrihemate (a form of free iron) has direct nephrotoxic effects, by catalysing free radical production (e.g. hydroxy radicals) resulting in oxidative cell injury (Vanholder *et al.*, 2000; Baxter and Moore, 2003). The haem centre of myoglobin also results in direct kidney injury by initiating lipid peroxidation (Vanholder *et al.*, 2000). It is also now evident that, using a rhabdomyolysis mice model, myoglobin released from damaged muscle may increase vasoconstriction within the renal afferent arterioles (Liu *et al.*, 2017). Eventually, multiple organ failure and death follow the myoglobin-induced acute kidney failure (La Grange *et al.*, 2010). Thus, rhabdomyolysis can be considered the primary malignancy in capture myopathy.

### Clinical signs and pathology

Classically, the initial clinical signs observed in animals suffering from capture myopathy are anxiety, shivering, rapid breathing, bent neck (*torticollis*), dark red urine and hyperthermia. In more protracted cases, animals may also present with lame or stiff limbs, appetite loss and constipation and can appear weak or lethargic. Once the animal presents with these signs, the probability of recovery is very poor (Wallace *et al.*, 1987; Spraker, 1993; La Grange *et al.*, 2010).

Although animals suffering from capture myopathy often present with these symptoms, there is a wide variation in their presentation, which has led to various authors classifying capture myopathy into different syndromes (Harthoorn, 1976; Spraker, 1993; La Grange *et al.*, 2010; Paterson, 2014). Harthoorn (1976) and Spraker (1993) each described four syndromes that closely resemble one another. The main difference, however, is that Harthoorn (1976) refers more to a time frame and associated clinical signs, whereas Spraker (1993) focuses more on clinical presentations (Harthoorn, 1976; Spraker, 1993; Montané *et al.*, 2002; Paterson, 2014). The four syndromes described are as follows.

#### Hyper acute or capture shock syndrome

Sudden death of a wild animal may occur during capture or up to a few hours thereafter (1 to 6 hours after capture). The animal may present with tachypnoea, tachycardia, a weak pulse, hyperthermia and lethargy and may even die. Macroscopic lesions on post-mortem include intestinal, hepatic and pulmonary congestion. Blood may be found in the lumen of the small intestine. On histopathology, multifocal areas of necrosis are evident in the brain, liver, adrenal glands, lymph nodes, spleen, pancreas, kidneys, heart and skeletal muscles (Harthoorn, 1976; Spraker, 1993). Laboratory serum biochemistry may illustrate elevated enzymatic activity for lactate dehydrogenase (LDH), CK and aspartate aminotransferase (AST) (Spraker, 1993; Paterson, 2014). Metabolic acidosis may be present, as indicated by a low blood pH (Harthoorn, 1976). The acidosis, if severe enough, results in electrolyte imbalances, causing cardiac fibrillation and death. However, haemolysis and muscle damage will result in hyperkalaemia and will affect normal neuronal conduction in the heart, leading to cardiac fibrillation and ultimately death. This fibrillation will be exacerbated if high circulating adrenalin from the adrenals is present (Harthoorn, 1976; Guis *et al.*, 2005).

This capture shock syndrome has a similar underlying pathophysiology to neurological vasogenic shock. The main distinguishing factor between the two is the presence of rhabdomyolysis in capture shock syndrome and the resultant myoglobin protein detected in the renal tubules, which is the main cause of the kidney injury (Vanholder *et al.*, 2000; Guis *et al.*, 2005; Oberem and Oberem, 2011). Distinguishing between these two conditions does not make them mutually exclusive. Additionally, it is considered that capture myopathy is a continuation of the initial shock condition. During the shock phase, kidney and muscle injury is believed to be initiated by ischaemic hypoxia. Surviving this phase, kidney injury in animals is compounded by the protein by-products of severe rhabdomyolysis (Spraker, 1993).

#### Acute or ataxic myoglobinuric syndrome

This syndrome is the most frequently observed and can occur hours to a few days after the capture event. The animals may show ataxia, torticollis and myoglobinuria in varying degrees. The same elevations in serum enzymes are seen as before (AST, CK and LDH), but blood urea nitrogen (BUN) is also elevated (Harthoorn, 1976; Spraker, 1993). Fortunately, an animal with mild symptoms may be better off surviving. On gross pathology, the kidneys are dark red and swollen, and the bladder may be empty or contain a small volume of red–brown fluid. In the flexor and extensor muscles of the limbs and the cervical and lumbar muscles, soft, pale and dry areas are observed with central white foci (severe muscle necrosis—a classical sign of capture myopathy) (Spraker, 1993; Roe and Spraker, 2012; Blumstein *et al.*, 2015).

The primary muscles affected are the quadriceps and the gastrocnemius muscles. The longer the animal survives, the more prominent is the muscle pathology (Harthoorn, 1976; Spraker, 1993; Meyer *et al.*, 2008). On histopathology, the main lesions are found in the kidneys and muscle. The kidney tubules appear dilated and necrotic, and myoglobin casts are present. The muscle fibres are swollen with striation loss, fragmentation of myofibrils and sub-sarcolemmal nuclear pyknosis; the latter representing an irreversible condensation of chromatin in the nucleus of a cell that is undergoing programmed cell death (Harthoorn, 1976; Spraker *et al.*, 1987; Wallace *et al.*, 1987; Montané *et al.*, 2002; La Grange *et al.*, 2010). The other distinguishing feature of this syndrome is that cardiac tissue is often severely affected, supporting a possible renaming of this syndrome to capture-induced cardiomyopathy (Fitte, 2017).

#### Sub-acute or ruptured muscle syndrome

Animals with this syndrome appear normal, but within 1 to 2 days after capturing clinical signs appear. They mostly die within a few days or may survive for a few weeks (Harthoorn, 1976; Spraker, 1993). The animals present typically with ruptured gastrocnemius muscles that result in dropped hindquarters and hyperflexion of the hocks (Harthoorn, 1976; Spraker, 1993; Paterson, 2014). Often, these animals are unable to stand, giving rise to tetraplegia. Torticollis is evident as a result of cervical muscle injury (Harthoorn, 1976).

The muscular lesions are similar to the ataxic myoglobinuric syndrome but are more severe and extended. Lesions also occur in the forelimb, diaphragm and cervical and lumbar muscles (Harthoorn, 1976; Lewis *et al.*, 1977; Spraker, 1993). Monophasic myonecrosis is the main histopathological finding in these muscles. Sarcolemma proliferation with muscular regeneration and fibrosis is more evident in this condition and used to distinguish between the sub-acute and chronic form of capture myopathy (Spraker, 1993; Paterson, 2014). LDH, CK and AST are markedly elevated, whereas BUN is either normal or slightly elevated (Harthoorn, 1976; Spraker, 1993; Paterson, 2014).

#### Chronic debility or delayed per-acute syndrome

This syndrome occurs rarely. Harthoorn (1976) also referred to this phase as the ‘indefinite phase’. Typically, these animals have been captured at least once in the past. When they are exposed to a second, usually mild stressful event (often another capture), death occurs within a few minutes (Montané *et al.*, 2002). The animals will try to escape but will suddenly come to a standstill, show pupillary mydriasis and die (Spraker, 1993). Lesions on gross pathology are mild with either a few or no pale necrotic areas visible in the muscles. However, histopathology does reveal rhabdomyolysis in skeletal muscle—typically in the hind limbs (Harthoorn, 1976; Chalmers and Barrett, 1977; Lewis *et al.*, 1977). The cardiac tissue also shows variable interstitial fibroses (Harthoorn, 1976). The underlying pathogenesis appears to be related to sudden hyperadrenalism that promotes a hyperkalaemic incident, disrupting cardiomyocytic cell membrane depolarization, resulting in fatal ventricular fibrillation (Montané *et al.*, 2002).

What is evident in the classification of the various phases or syndromes is that muscle rhabdomyolysis plays a central role in the pathological presentation and outcome of capture myopathy.

## Treatment of capture myopathy

Currently, there is no treatment that ensures recovery from capture myopathy. The most successful approach is adopting good preventative practises (Businga *et al.*, 2007; Roe and Spraker, 2012). The use of tranquillizers during and after capture has aided in reducing the occurrence of capture myopathy but remains anecdotal at best. It has, however, not eliminated capture myopathy-related deaths due, in part, to human factors and capture methods employed (La Grange *et al.*, 2010). For example, it is well known that if field staff members who are not adequately trained in the correct handling or restraint of a specific species this may pose a risk to these animals. Also, cost constraints may eliminate the option of using a helicopter, or a veterinarian with access to capture drugs, which may result in a capture operator opting to use less than optimal methods for capture and translocation purposes. Furthermore, if appropriate capture methods are not performed correctly by well-trained staff who have an in-depth knowledge of the species in question, it may result in increased mortalities. Additionally, failure to recognize and record capture myopathy deaths may have a negative impact on accurate reporting of its prevalence.

Capture myopathy therapy consists mainly of supportive treatment with anecdotal successes reported following some intensive efforts (Spraker, 1993; Smith *et al.*, 2005; Businga *et al.*, 2007). Most success in treatment seems to be achieved in animals that develop the acute or ataxic myoglobinuric syndrome. The treatment of the sub-acute or ruptured muscle syndrome is symptomatic and protracted. By its nature, the prognosis for animals with this syndrome is very poor due to the severe, often permanent, vital tissue injury that occurs. In the hyper-acute or capture shock syndrome there is often not enough time to initiate treatment, and due to the severity of the pathophysiology, it is difficult to implement effective treatment. The only advice that can be provided in the delayed or chronic syndrome is that animals are handled gently (stress free) after capture events (Harthoorn, 1976).

Current therapies that are used for the treatment of capture myopathy and other conditions causing rhabdomyolysis are summarized in Table 1. The efficacy of many of these therapies has not been validated, and most of these therapies are difficult to apply practically under field conditions. Unfortunately, once an animal shows clinical signs of capture myopathy, especially the appearance of dark coloured urine, the prognosis is poor. This highlights the need for proper clinical trials in capture myopathy.

**Table 1:** A summary of reported and/or proposed treatment regimens for capture myopathy and other forms of rhabdomyolysis

**Treatment management regime**	**Practicality**	**Evidence of success**	**References**

***Analgesics***			
Opioids To alleviate any pain the animal may experience due to capture myopathy	Easy to administer; expensive	No clinical trials or studies exist indicating its efficacy or success in treating capture myopathy.	Spraker (1993), Paterson (2014)

***Inhibitors of inflammation***			
Corticosteroids and non-steroidal anti-inflammatory medication To inhibit the inflammatory response and act as an analgesic	Relatively cheap	Multiple drug combinations including other treatment showed some improvements in birds. A dog with rhabdomyolysis recovered using a combination of treatments. No clinical trials or studies exist indicating its efficacy or success in treating capture myopathy.	Spraker (1993), Wells *et al.* (2009), Ward *et al.* (2011), Paterson (2014)

***Chemical inhibition of muscle contraction***			
Dantrolene Is a drug registered for treatment of malignant hyperthermia Acts directly on the ryanodine receptor to prevent calcium release from the sarcoplasmic reticulum	Very expensive, sensitive to light, poor solubility in water and large quantities required in large animals May cause adverse effects including muscle weakness, hepatoxicity and neurological impairment	Used successfully in the treatment of neuroleptic malignant syndrome and spasticity in humans. Some success reported to prevent recurrent exertional rhabdomyolysis in horses. A dog with rhabdomyolysis recovered completely using a combination of treatments. No direct benefit in treatment of exertional heatstroke in humans. No clinical trials or studies exist indicating its efficacy or success in treating capture myopathy.	Watson *et al.* (1993), Edwards *et al.* (2003), McKenzie *et al.* (2004), Wells *et al.* (2009), Paterson (2014), Schneiderbanger *et al.* (2014), Choi *et al.* (2017)

***Correction of acidosis***			
Sodium bicarbonate Solution of NaHCO_3_ in saline Infusion titrated against blood pH To maintain circulatory volume and alleviate metabolic acidosis, hyperkalaemia and myoglobinuria	Relatively inexpensive Titration against blood pH values is very difficult, time-consuming and expensive Very difficult to perform in field conditions	Treatment of wild zebra (*Equus zebra*) after capture 9 from 12 treated animals survived. Untreated animals all succumbed to capture myopathy. Post-mortem signs were typical of capture myopathy. Immediate alleviation of cardiac dysfunction and dyspnoea. Bicarbonate treatment for acidosis lost favour over the years. Other cases reported no effect on capture myopathy outcome.	Harthoorn *et al.* (1974), Forsythe and Schmidt (2000), Bagley *et al.* (2007), Wells *et al.* (2009), Paterson (2014)
Fluid therapy Saline or ringers lactate infusion	Relatively inexpensive	Although frequently used to prevent kidney damage in human and equine rhabdomyolysis, no clinical studies exist that determined its efficacy in wildlife with capture myopathy.	Bagley *et al.* (2007), Valberg (2009)

***Nutritional support and antioxidant supplementation***			
Added nutritious feed during theprotracted treatment andrehabilitation process of animalsrecovering from capture myopathy Intravenous or oral administration ofantioxidant compounds such as vitamin E,selenium,co-enzyme Q10 andl-carnitine	Relatively inexpensive May have adverse effects in high doses	Although some success was reported in human metabolic myopathies, no direct evidence or controlled studies exist to suggest efficacy of any antioxidant supplementation or nutritious feed to treat capture myopathy.	Chalmers *et al.* (1979), Graffam *et al.* (1991), Beech (1997), Rogers *et al.* (2004), Smith *et al.* (2005), van Adel and Tarnopolsky (2009), Wells *et al.* (2009); Parikh *et al.* (2009); Ward *et al.* (2011), Montgomery *et al.* (2011); Landau *et al.* (2012), Valberg (2016)

***Anxiety alleviation***			
Anxiolytic (e.g. benzodiazepines and some tranquilisers) To reduce muscular rigidity and any further stress	Relatively inexpensive;possible side effects	No clinical studies or evidence exist to suggest that it successfully treats capture myopathy.	Ward *et al.* (2011); Wolfe and Miller (2005)

***Hyperbaric oxygen supplementation***			
Drug-induced rhabdomyolysisin a human. Successfully treated with the conjunctive use of hyperbaric oxygen	Impractical in animals in field conditions; concern of oxygen toxicity	No clinical trials exist to prove its efficacy in wildlife suffering from capture myopathy	Abdullah *et al.* (2006), Parikh *et al.* (2009)

***Cooling***			
Ice water immersion and water dousingwith or without fanning, infusionof cold saline solution Alleviating hyperthermia in humans and animals with exertional heatstroke or hyperthermia	Inexpensive Can be time-consuming and may take a long time for temperature to reach normality If hyperthermia is not diagnosed and treated adequately morbidity and mortality can occur Some methods are impractical in the field	Efficacy of cold water immersion has been proven in case studies of humans with exertional heatstroke. No clinical trials exist to show that cooling can successfully treat or prevent capture myopathy.	Arnemo and Caulkett (2008), Rae *et al.* (2008), Sawicka *et al.* (2015)

## Prevention of capture myopathy

The best measure to prevent capture myopathy is to ensure that animals are limited to external causes of stress. Although not yet clinically tested in controlled settings, the preventative measures include utilising the correct capture method, adequate planning, the use of tranquilizers and immobilization drugs, habituation, cooling of animals and the environment (e.g. time of day and temperature).

### Capture method

Deciding on whether to use chemical or physical restraint and which type of these restraints to use, is a decision that will be dictated by economics, level of operator experience, ethics, environment and the species to be captured.

For chemical restraint, the type of immobilization drugs used is determined by the species to be captured, availability of the immobilization drugs, route of administration and the delivery system (oral, pole syringe or dart gun). Failure of chemical restraint and subsequent tranquilization will lead to an increased stress response, which can result in trauma and possibly even capture myopathy. This failure results from equipment and darting failure, lack of preparation and planning, poor staff communicationand execution and poor selection and under-dosing of immobilization drugs (Spraker, 1993; La Grange *et al.*, 2010; Atkinson *et al.*, 2012).

It is possible to restrain animals physically with or without tranquilization. Due to the hands-on nature of this type of technique, adequate experience of an operator is essential to ensure that animals are handled in a way that minimize the stress response. Different methods tailored to the operation at hand are used in the field and include

- Direct physical restraint—mostly appropriate for small birds and reptiles and not suited for use in larger free-roaming wildlife.
- Ropes—often utilized to restrain and manipulate animals but can cause stress and trauma to both animal and handler if not used appropriately.
- Drive nets or drop nets—preferred for mass capture of smaller antelope. Excitable species might quickly succumb; therefore rapid tranquillization is necessary to calm animals.
- Net gun—used for individual animals and requires a skilled and trained operator to ensure safety of the animal.
- Physical barriers—erecting a fence or shaded netting to manipulate the directional movement of animals into large bomas is often used in mass capture operations (Grange and La Grange, 2006; La Grange *et al.*, 2010; Atkinson *et al.*, 2012).

### Adequate planning of the capture process

The primary goal of the capture process should be to reduce stress during the operation through adequate risk assessment and contingency planning (Grange and La Grange, 2006). In particular, the duration that animals are exerted, handled and transported should be minimized. Achieving these goals requires significant resources and planning that include reduction of the time spent gathering, loading, restraining or immobilizing and transporting of the animals (Grange and La Grange, 2006; La Grange *et al.*, 2010; Atkinson *et al.*, 2012). A qualified experienced capture team with adequate knowledge of the specific species being captured is vital to reduce stress and trauma to the animals (Atkinson *et al.*, 2012). These practices reduce the incidence but do not eliminate the occurrence of capture myopathy. It raises an important question as to why certain species or specific individual animals within the same species are more susceptible to developing capture myopathy than others (Meyer *et al.*, 2008; La Grange *et al.*, 2010; Mason, 2010; Atkinson *et al.*, 2012).

### The use of tranquilisers and immobilization

The administration of tranquilizers, sedatives and sometimes immobilizing drugs during capture procedures are recommended practices that appears to decrease the incidence of capture myopathy and improves survival rates. However, the use of drugs should not be a substitute for poor planning, lack of habituation or poor handling techniques of animals (La Grange *et al.*, 2010). In fact, the use of these drugs has no effect on reducing the inevitable increase in body temperature of captured animals, and if prolonged, this hyperthermia may contribute to the development of capture myopathy (Meyer *et al.*, 2008).

Immobilizing drugs can also disrupt thermoregulation. Opioids (immobilizer) and α_2_-agonists (sedative) are known to alter thermoregulation and may contribute to the capture-induced hyperthermia (Fahlman *et al.*, 2008; Meyer, 2009). Although not yet understood, opioids are thought to cause hyperthermia through sympathetic stimulation, which increases metabolism and resultant heat production (Meyer, 2009; Atkinson *et al.*, 2012). Furthermore, many of the drugs that are used in wildlife like ketamine, haloperidol, diazepam, naltrexone and succinyl choline are known to induce rhabdomyolysis directly in humans (Guis *et al.*, 2005).

The use of long-acting tranquilizers post-capture has shown great benefit by lowering the incidence of capture myopathy and mortalities from >20% to <2% (La Grange *et al.*, 2010). However, these drugs need to be used with caution as cases of overdosing can result in unwanted side effects, such as anorexia and extrapyramidal signs (Atkinson *et al.*, 2012). Extrapyramidal effects cause an increase in uncontrolled muscular activity and thus may play a role in the development of capture myopathy.

### Habituation

Before a capture is executed, it has been recommended that, wherever possible, wild animals should be exposed to the presence of humans and motorized vehicles (habituated) to attempt to reduce the stress response to capture. Animals should also be allowed adequate time to habituate to a new environment before repeated handling events (La Grange *et al.*, 2010). For some species, habituation before minor procedures such as vaccinations, venepuncture and physical examinations can be adequate to allow for no or minimal restraint (Meyer *et al.*, 2008; Paterson, 2014). For this degree of habituation and reduction in stress response to occur, continuous gradual desensitization is required but is very difficult to attain in free-living wild animals.

### Cooling

Hyperthermia is believed to play a role in the development of capture myopathy. Therefore, it has become a common practice to attempt to actively cool animals that develop capture-induced hyperthermia (Meyer, 2009). This practice originates from knowledge gained from classic and exertional heatstroke (Rae *et al.*, 2008). If core temperatures surpass 41°C, the individual is submerged in an ice bath where after active fanning may be required until the body temperature returns to normal. The duration of hyperthermia, and not its magnitude, is thought to be the main differentiating factor in the outcome of heatstroke in humans. Hence, effective and rapid cooling is required (Casa *et al.*, 2012). The adverse impact of the duration and magnitude of hyperthermia is still to be determined in captured wildlife.

Despite not knowing the actual consequences of capture-induced hyperthermia, many methods are employed to cool hyperthermic captured wild animals, varying from water dousing, ice packing, cold intravenous fluid administration, mist sprayers and the use of cold water enemas (Sawicka *et al.*, 2015). Water dousing seems the most practical and effective method of cooling hyperthermic animals in the field (Sawicka *et al.*, 2015). Although effective cooling did not protect against or prevent any of the associated pathophysiological changes that were induced in captured blesbok (*Damaliscus pygargus phillipsi*), the findings suggest that the contribution of capture-induced hyperthermia may be less than previously thought (Fitte, 2017).

### Environmental conditions

A customary recommendation to prevent capture myopathy or limit hyperthermia-associated complications is to avoid capture operations on hot days or during the time of day when temperatures are high. The prescribed recommendation is not to capture wild animals when the ambient temperature exceeds 25°C (Atkinson *et al.*, 2012). These recommendations may stem from the belief that capture myopathy is similar to the human condition of classic heatstroke, where hot ambient conditions are the primary catalyst (Rae *et al.*, 2008; Meyer, 2009).

Additionally, the clinical presentation of capture myopathy is like that of exertional rhabdomyolysis and exertional heatstroke found in horses and humans, respectively, where hyperthermia and muscle rhabdomyolysis are primary symptoms. However, in-depth analyses of humans suffering from exertional heatstroke concluded that ambient conditions were not associated with the elevated body temperatures and that excessive production of endogenous heat within individuals was more likely the cause (Rae *et al.*, 2008). A similar conclusion was drawn where the magnitude of capture-induced hyperthermia in impala (*Aepyceros melampus*) was not associated with environmental conditions or the intensity and amount of exertion during the capture process, but rather the stress response itself (Meyer *et al.*, 2008). Furthermore, cooling did not prevent or reduce the capture-induced pathophysiological effects, suggesting that the role of capture-induced hyperthermia in the pathogenesis of capture myopathy may be overplayed (Fitte, 2017).

Although all these preventative measures are implemented, capture myopathy still arise, is unpredictable and highlights a lack of understanding of the biological mechanisms that cause this condition. Fortunately, several conditions with similar symptoms and pathophysiology exist that may assist in studying capture myopathy.

## Similar myopathy conditions in mammals

Many conditions presenting with hyperthermia and rhabdomyolysis exist in mammals, but each with a different causation. These include among others exertional heatstroke, crush injury, malignant hyperthermia and exertional rhabdomyolysis.

### Exertional heatstroke in humans

Exertional heatstroke may occur after excessive exercise in unfit humans, and rarely in highly trained athletes. It has also been termed ‘march myoglobinuria’ as it is a prominent condition presenting after severe exertion in the first 6 months of basic military training in new recruits (Spraker, 1993; Rae *et al.*, 2008; Capacchione and Muldoon, 2009; Landau *et al.*, 2012). The condition is rare but, once present, often fatal. Although hyperthermia occurs in this condition, it should be distinguished from classical or environmental associated heatstroke. The latter results from inefficient thermoregulatory control when individuals are subjected to high environmental temperatures and occurs mostly in the very young and old. Secondly, patients with classic heatstroke do not sweat, whereas in exertional heatstroke, patients sweat profusely (Rae *et al.*, 2008).

Poor heat dissipation from endogenous heat production is believed to cause exertional heatstroke (Smith, 2005). The prominent symptoms are elevated core body temperatures (>41°C), myalgia, muscle weakness (caused by muscle rhabdomyolysis) and dark red-coloured urine (myoglobinuria). The exact mechanism that triggers the pathophysiology is still unclear (Baxter and Moore, 2003; Smith, 2005). Some risk factors have been associated with this condition and include dehydration, concurrent illness, sleep deprivation, obesity, alcohol consumption, poor fitness and excessive clothing (Guis *et al.*, 2005; Smith, 2005). These are all difficult to reproduce or study in humans.

A puzzling occurrence is that exertional heatstroke can occur in healthy fit individuals that are prepared and conditioned for the event that they are participating in. This perplexity poses the question whether these individuals may be predisposed to developing exertional heatstroke by a mechanism that is not associated with fitness or health (Rae *et al.*, 2008; Capacchione and Muldoon, 2009). Importantly, it is quite normal for elite endurance runners to develop core body temperatures >40°C without developing any abnormalities. They can therefore tolerate excessive heat better than untrained individuals, and this exercise-induced hyperthermia is most likely not the inciting cause of exertional heatstroke (Wyndham, 1977; Marino *et al.*, 2004). Some of the underlying factors that may precipitate rhabdomyolysis in this condition may include abnormalities in metabolism e.g. glycogen storage diseases, diabetic ketoacidosis, mitochondrial myopathies, hypokalaemia and hypophosphataemia. Other proposed causes include muscular parasitosis (trichinellosis) and toxins. Chronic medication, the ingestion of over-the-counter drugs (e.g. non-steroidal anti-inflammatories) to improve performance, or illicit drug use have also been associated with the development of rhabdomyolysis and, potentially, exertional heatstroke (Watson *et al.*, 1993; Baxter & Moore, 2003).

Like capture myopathy, treatment for exertional heatstroke is limited and usually involves rapid cooling by submerging the patient in ice water. However, if the diagnosis and treatment is delayed, this treatment can be unsuccessful (Baxter and Moore, 2003). The hyperthermia that occurs during exertional heatstroke episodes usually continues after exercise has ceased, indicating that the origin of the heat is not from contracting muscles but from yet unknown ‘heat generators’ (Rae *et al.*, 2008).

Many similarities between the ataxic myoglobinuric syndrome of capture myopathy in animals and exertional heatstroke in humans exist. These similarities include hyperthermia, rhabdomyolysis and myoglobinuria. It may be that the suspected triggers and predisposing factors for these conditions are similar, but poorly understood or researched (Bartsch *et al.*, 1977).

### Compartment syndrome and crush injury rhabdomyolysis

Compartment syndrome may occur after severe trauma like crush injury or fracture(s). It results in an increased interstitial pressure within a closed osteofascial compartment that limits local circulation. This latter effect results in ischaemic damage, which, if prolonged, can cause irreversible damage. Compartment syndrome is believed to contribute towards rhabdomyolysis and *vice versa*. It has also been implicated in the pathophysiology of capture myopathy. Whether acute kidney injury follows is most likely dependent on the extent of the rhabdomyolysis (Baxter and Moore, 2003; Malinoski *et al.*, 2004).

Rhabdomyolysis caused by crush injury is well documented in humans, especially in victims of car accidents and earthquakes. The presenting pathophysiology is similar to exertional heatstroke, except that the inciting cause is external trauma to the muscles (Malinoski *et al.*, 2004).

### Malignant hyperthermia

A well-known genetic defect primarily described in humans and pigs causes a condition known as malignant hyperthermia (Wappler, 2001). It stems from a mutation in the ryanodine 1 receptor (RYR1), with its function to regulate Ca^2+^ release from the sarcoplasmic reticulum (Wappler *et al.*, 2001). To date, approximately 30 mutations in the over 300 variations of the RYR1 gene have been associated with causing malignant hyperthermia in humans (Schneiderbanger *et al.*, 2014). The mutation makes the RYR1 very sensitive to certain triggers, like caffeine and general anaesthetic agents (e.g. halothane). The triggers cause uncontrolled release of Ca^2+^ from the sarcoplasmic reticulum into the muscle fibres resulting in prolonged muscle contraction. This contraction results in a hypermetabolic state that presents with increased aerobic and anaerobic metabolism, leading to hypoxia, metabolic acidosis, increased CO_2_ production and hyperthermia. In addition, sarcoplasmic Ca^2+^ resorption utilizes vast amounts of adenosine triphosphate (ATP), depleting intracellular ATP, phosphocreatine and glycogen stores. These effects result in protracted rigidity and rhabdomyolysis (Wappler, 2001; Schneiderbanger *et al.*, 2014).

Volatile halogenated inhalation anaesthetics and the muscle relaxant succinylcholine are the most common triggers of malignant hyperthermia. Exposure does not always trigger the condition, and it is postulated that the condition might be dose dependent (Capacchione and Muldoon, 2009; Schneiderbanger *et al.*, 2014). However, malignant hyperthermia does not necessarily need to be drug induced. Severe emotional or physical stress can also be a trigger and has been well described in pigs (Wappler *et al.*, 2001; Schneiderbanger *et al.*, 2014).

Malignant hyperthermia in pigs is also known as porcine stress syndrome, which causes an economically important condition called pale, soft, exudative meat. A single mutation in the RYR1 gene is the cause for this syndrome in all pig breeds, and animals present with muscle rigidity, acidosis and hyperthermia (Fujii *et al.*, 1991; Moochhala *et al.*, 1994). Rapid glycolysis, increased lactate formation and muscle necrosis result in pale areas of skeletal and heart muscle—thus giving rise to the name ‘Pale Soft Exudative’ meat syndrome (Mitchell and Heffron, 1980). Identified triggers include high environmental temperatures, exertion, fighting, mating and parturition (Mitchell and Heffron, 1982). Its aetiology and presentation are very similar to that of capture myopathy. Therefore, as in pigs, Mitchell and Heffron (1980) suggested that a genetic anomaly may be present that result in an abnormal response to stress that causes this condition to occur in wildlife.

A small cohort of clinical studies has proposed an association between exertional heatstroke and malignant hyperthermia in humans (Schneiderbanger *et al.*, 2014). In a controlled study by Wappler *et al.* (2001), 12 patients who survived exertional heatstroke were tested with the standard European test for malignant hyperthermia known as the ‘in vitro contracture test’. This test involves exposing an excised piece of muscle tissue to various concentrations of halothane and caffeine. Ten of the twelve patients tested positive, one showed an equivocal result and another was negative for the condition (Wappler *et al.*, 2001). Therefore, a mutation in the RYR1 receptor is very likely to make individuals acquire exertional heatstroke. The mutation causing malignant hyperthermia has also been found in horses and dogs, but too few studies have been performed in wildlife to conclude its presence (Aleman, 2008). One study did attempt to determine if capture-induced hyperthermia and capture myopathy could be associated with malignant hyperthermia by subjecting muscle specimens from four black-tailed deer to the in vitro contracture test. All specimens were negative, indicating no association (Antognini *et al.*, 1996). However, the small sample size and the fact that healthy animals, and not animals that developed capture myopathy, were tested make the findings from this study inconclusive. Therefore, it is imperative that this area in capture myopathy be reopened for investigation.

### Exertional rhabdomyolysis in horses

Exertional rhabdomyolysis is a popular condition in horses, frequently diagnosed, and known by many names, such as tying-up, set fast, Monday-morning disease, azoturia, chronic intermittent rhabdomyolysis and equine rhabdomyolysis syndrome. Horses that partake in Polo Cross have the highest incidence of acquiring exertional rhabdomyolysis (~13%), with thoroughbreds competing in horse racing having a much lower incidence (~6%) (Beech, 1997; Aleman, 2008; Valberg, 2009). Anecdotal risk factors to this condition include being two (2) years of age, female, a highly strung nature, continuous exercise with minimal rest days, high concentrate carbohydrate diets and lameness before the event (McGowan *et al.*, 2002).

Clinical presentation of this condition is muscle stiffness, tachypnoea, sweating, painful hindquarter muscles and the reluctance to move. The diagnosis is made on history of exercise, elevated serum CK and AST concentrations. Moderate to severe rhabdomyolysis may result in myoglobinuria, metabolic alkalosis and azotaemia due to the effects of the myoglobin on the kidneys (Beech, 1997; Valberg, 2009).

The condition can be divided into two syndromes: (i) horses that develop a sporadic episode of exertional rhabdomyolysis and (ii) chronic exertional rhabdomyolysis, where it occurs frequently, and the horses appear to have an underlying susceptibility. Triggers of sporadic exertional rhabdomyolysis are believed to include excessive exertion, heat exhaustion and electrolyte imbalances. Glycogen storage myopathies and nutritional imbalances have also been associated with the manifestation of chronic exertional rhabdomyolysis (Lentz *et al.*, 1999; Valberg, 2009).

#### Polysaccharide storage myopathy

Stephanie Valberg and colleagues first described polysaccharide storage myopathy (PSSM) in 1992, when a cohort of horses with recurrent exertional rhabdomyolysis tested positive for abnormal glycogen depositions in their muscle biopsies (Valberg *et al.*, 1992). Over the years, PSSM was found to be mainly prevalent in Quarter horses (between 6% and 12%) and draught horses (36% of Belgian draught horses) (Aleman, 2008; McCue *et al.*, 2008). The condition manifests in horses when exercised and results from the inability of their muscle fibres to utilize glycogen for ATP synthesis. The cause of PSSM has been attributed to a mutation in the gene that encodes for the muscle glycogen synthase enzyme, *Gys1* (McCue *et al.*, 2008). The gold standard for diagnoses of PSSM is the amylase periodic acid Schiff (PAS) stain on histologically prepared sections. A muscle fibre that harbours the mutation would reveal granular precipitate in type II muscle fibres, indicating the presence of abnormal glycogen (Aleman, 2008; Sierra *et al.*, 2012). Additional mutations in conjunction with the *Gys1* mutation may aggravate the symptoms of PSSM (McCue *et al.*, 2009).

A condition with the same histological presentation as PSSM has been found in the muscle of 11 species of aquatic mammals (cetaceans) (Sierra *et al.*, 2012). Out of 148 beached cetaceans, PAS positive, diastase-resistant inclusions were found in 26 of these animals, being consistent with abnormal glycogen deposits or complex polysaccharide. In addition, these inclusions also stained positive for ubiquitin, with type II fibres specifically affected. Whether this condition is also caused by a glycogen synthase mutation still needs to be established (Sierra *et al.*, 2012). Thus, there could be a link between PSSM and capture myopathy, warranting further investigation (Roe and Spraker, 2012).

#### Other unknown causes of recurrent exertional rhabdomyolysis

Recurrent exertional rhabdomyolysis in horses is diagnosed when a horse that develops the condition tests negative for the *Gys1* mutation associated with PSSM, as well as the RYR1 mutation associated with malignant hyperthermia. Histologically, the muscle fibres of these horses show no sign of excessive or abnormal glycogen storage, but these horses have vast numbers of fibres with central nuclei (Lentz *et al.*, 1999; Aleman, 2008; Valberg, 2009). Recurrent exertional rhabdomyolysis is frequently found in thoroughbred horses with an average prevalence of 5–10%. During the racing season, up to a fifth of horses may develop this type of rhabdomyolysis and the cause is believed to be a genetically predisposition associated with abnormal intramuscular Ca^2+^ regulation (Aleman, 2008). The involvement of important defects in RYR1, such as the dihydropyridine receptor-voltage sensor and sarcoplasmic reticulum calcium ATPase, has been excluded as the triggers of this condition. However, these horses do test positive using the in vitro Contracture test (Aleman, 2008). Thus, another mechanism for abnormal muscle Ca^2+^ regulation may exist in these animals and determining this mechanism may reveal insights into why some wildlife develop capture myopathy.

To summarize, there are stark similarities between known rhabdomyolysis and hyperthermic conditions in humans, domestic animals and other species. Studying their similarities can guide research aimed at unravelling the pathomechanisms and causes of capture myopathy.

## Hypothetical causes of capture myopathy

Capture myopathy has been proposed to be an ‘inherent mechanism’ that assists wild animals to ‘die’ quicker when caught by a predator and indirectly assists the predator in conserving energy (Spraker, 1993). However, this theory seems highly unlikely. For example, a predator would never chase wild prey for prolonged periods of time in their natural habitat, whereas in a capture situation, the chase might be considerably longer and involve more stressors (La Grange *et al.*, 2010). Additionally, many animals have escaped the jaws of their predators and subsequently survived. Thus, the concept of accelerated death when caught by a predator contradicts the widely accepted theory of ‘survival of the fittest’, as prey will never evolve mechanisms to assist its predator in conserving energy. Furthermore, the theory of natural selection postulates that an evolutionary adaptation of a trait requires the continued reproduction of such a trait. ‘Dying quicker’ does not assist in transferring any traits to any future generations and would result in their extinction. Some predators, like wild dogs, may use exertional myopathy to their advantage since these animals are renowned for chasing prey over long distances, inducing exhaustion of their prey to complete the kill (Bartsch *et al.*, 1977). Similarly, indigenous humans of Southern Africa, known as the Koi San, are known for tracking and chasing a single animal to the point of exhaustion to get closer to the animal for bow and arrow shots (Liebenberg, 2006). It is more conceivable that prey species have evolved physiological mechanisms in their muscles that aid the animal in escaping predation during the fight and flight response. Thus, the outcome of these mechanisms is a successful escape if the chase is of short duration, but the disadvantage is that the probability is high for these mechanisms to fail when over exerted and manifests as capture myopathy (Bartsch *et al.*, 1977).

The stress experienced by wild animals appears to be one of the key precipitating factors of capture myopathy (La Grange *et al.*, 2010). However, as is witnessed with malignant hyperthermia and recurrent exertional rhabdomyolysis, factors other than stress can trigger the development of rhabdomyolysis (Lentz *et al.*, 1999; Capacchione and Muldoon, 2009). Even by minimizing the stress response, some wild animals still develop capture myopathy (La Grange *et al.*, 2010). The stress-induced pathophysiological events that lead to capture myopathy are just not that well understood.

Old hypotheses have since been refuted for exertional heatstroke and capture myopathy. For example, the intensity and duration of exercise performed or high ambient temperatures during endurance events, or both, have been considered as primary risk factors to develop hyperthermia and exertional heatstroke in humans (Rae *et al.*, 2008). The same factors were extrapolated to causing hyperthermia and capture myopathy in wildlife. However, evidence from studies has since questioned these claims (Meyer *et al.*, 2008; Rae *et al.*, 2008). In fact, Meyer *et al.* (2008) showed that neither environmental temperature, the level of exertion nor the use of different drugs was associated with the extent of hyperthermia that developed during the capture of impala (Meyer *et al.*, 2008; Meyer, 2009). Rae *et al.* (2008) supported these findings in humans. Exertional heatstroke cases were reported at ambient temperatures as low as 4°C at low exercise intensities of short duration and distance (e.g. one athlete acquired exertional heatstroke after only 2 km of running at 7.4 km/h for only 16 minutes at an ambient temperature of 16.7°C) (Rae *et al.*, 2008).

The above clearly indicates some underlying condition that induces excessive endothermy, of which the mechanisms is not yet understood (Bartsch *et al.*, 1977; Smith, 2005; Rae *et al.*, 2008; Capacchione and Muldoon, 2009). Therefore, many external factors, like high ambient temperatures, may only be playing a secondary or aggravating role. Additionally, why certain species and individual animals are more prone to the development of capture myopathy is still not known, but suggest an inherent genetic predisposition (Antognini *et al.*, 1996; La Grange *et al.*, 2010; Mason, 2010). The proposed causes discussed below should also be contextualized with the response to fear and capture, as reviewed previously.

### Inherent predisposition to capture myopathy

#### Species and size

Although numerous vertebrate species can be affected by capture myopathy, mammal and bird species seem most frequently affected, fish and amphibians less so and only a few cases have been reported in reptiles (Spraker, 1993; Phillips *et al.*, 2015). Roan (*Hippotragus equinus*), nyala (*Tragelaphus angasii*), tsessebe (*Damaliscus lunatus*), red hartebeest (*Alcelaphus buselaphus caama*), springbok (*Antidorcas marsupialis*), kudu (*Tragelaphus strepsiceros*) and giraffe (*Giraffa camelopardalis*) are considered some of the most susceptible African ungulate species to capture myopathy (Oberem and Oberem, 2011). In North America, the condition has been observed in a few species including white-tailed deer (*Odocoileus virginianus*) (Beringer *et al.*, 1996; Dechen Quinn *et al.*, 2014), black-tailed deer (*Odocoileus hemionus columbianus*) (Antognini *et al.*, 1996), pronghorn (*Antilocapra americana*) (Chalmers and Barrett, 1977) and elk (*Cervus elaphus*) (Lewis *et al.*, 1977). Southern chamois (*Rupicapra pyrenaica*) (López-Olvera *et al.*, 2007) and roe deer (*Capreolus capreolus*) (Montané *et al.*, 2002) are the most frequent European species affected by capture myopathy. Wild turkeys (*Meleagris gallopavo*) (Spraker *et al.*, 1987), sandhill cranes (*Grus canadensis*) (Businga *et al.*, 2007), rheas (*Rhea americana*) (Smith *et al.*, 2005), bar-tailed godwits (New Zealand) (*Limosa lapponica*) and long-legged shore birds are some of the recorded cases of capture myopathy in bird species (Rogers *et al.*, 2004; Blumstein *et al.*, 2015).

Marine animals are not excluded from acquiring capture myopathy, especially various whale species like finned pilot whale (*Globicephala melas*), Risso’s dolphin (*Grampus griseus*), pygmy sperm whale (*Kogia breviceps*) and Blainville’s beaked whales (*Mesoplodon densirostris*). The dolphin species in which capture myopathy was found include the false killer whale (*Pseudorca crassidens*), striped dolphin (*Stenella coeruleoalba*), Atlantic spotted dolphin (*Stenella frontalis*), spinner dolphin (*Stenella longirostris*) and bottlenose dolphin (*genus Tursiops*). The often poor success rate of cetacean rehabilitation is frequently attributed to stress-associated myopathies (Roe and Spraker, 2012; Sierra *et al.*, 2012; Herráez *et al.*, 2013). PSSM may play a significant role in cetaceans that develop rhabdomyolysis, suggesting a possible genetic cause of capture myopathy in these animals (Roe and Spraker, 2012; Sierra *et al.*, 2012; Herráez *et al.*, 2013). What was not clear from the literature was whether these aquatic mammals also presented with hyperthermia. Nevertheless, although certain species seem more susceptible and, hence, may have a genetic predisposition, it does seem evident that any animal can acquire and succumb to capture myopathy (Mason, 2010).

With large stranded cetaceans prolonged muscle compression may contribute to the rhabdomyolysis and myoglobinuric nephrosis that follows (Herráez *et al.*, 2007). This effect is also a recognized complication in rhabdomyolysis of large muscle masses in humans and may also increase the likelihood of renal failure in these individuals (Knochel, 1982). It is well known that immobilized large animals, such as rhinoceros, run the risk of rhabdomyolysis due to reduced blood flow and hypoxaemia (i.e. ischaemia), especially in their limbs, during recumbency (Meyer *et al.*, 2015; Cole *et al.*, 2017). Sadly, the incidence of rhinoceros that have been chemically immobilized by poachers and not killed has increased over the years, particularly due to this type of poaching method being more discreet compared to the noise of gunshots. These animals are often deserted without reversing the immobilizing drugs (most often opioid anaesthetic drugs) and may remain immobilized for hours before they are found or when the anaesthesia wears off (Meyer *et al.*, 2015; Cole *et al.*, 2017). The consequence is usually extensive myopathy, characterized by rhabdomyolysis and myoglobin-induced kidney injury, which carries a poor prognosis for many of these animals (Meyer *et al.*, 2015).

#### Age and physical condition

Young and old animals are reported to be more prone to develop capture myopathy, but the reasons for this anomaly are still unclear. Susceptibility to the condition seems to be increased by poor physical condition or being overweight (Harthoorn, 1976; La Grange *et al.*, 2010). The former state is commonly found in young and old animals, since these age groups are often of the lowest social rank in a herd. Interestingly, obesity seems to play an important role in predisposing humans to exertional heatstroke (Casa *et al.*, 2012). These conditional factors have merely been associated with capture myopathy and their specific roles need further investigation.

#### Skeletal muscle composition: fibre type, metabolism and oxidative stress defence

An alternative cause may be related to metabolism. Exercise increases the metabolic demand for ATP synthesis from aerobic and anaerobic metabolism of glucose and glycogen through glycolysis and the Krebs cycle and from fats through β-oxidation (Hawley and Hopkins, 1995). The metabolism of skeletal muscles from wild animals differs substantially from that seen in humans. Antelope species, such as springbok, kudu, mountain reedbuck (*Redunca fulvorufula*) and black wildebeest (*Connochaetes gnou*), have muscle with very high mitochondrial numbers and high oxidative capacities, equating to capacities found in highly trained human endurance athletes (Curry *et al.*, 2012; Kohn, 2014). Additionally, these animals (including some wild felid species) also have an enormous glycolytic capacity to metabolize glucose and glycogen through their glycolytic pathway to either feed into the Krebs cycle or to produce lactate (Kohn *et al.*, 2011; Curry *et al.*, 2012). It was also shown that individual muscle fibres from wild felids produce three times more power than their human equivalent, indicating the large demand for ATP from these metabolic pathways once muscle contraction commences (Kohn and Noakes, 2013).

Although not yet measured, it is postulated that during a fight or flight episode, the muscles of these animals possess the capacity to generate enormous quantities of ATP (Kohn *et al.*, 2011; Kohn, 2014). In stressed animals, β_2_-adrenergic receptor stimulation by adrenalin results in the production of cyclic AMP, which in turn increases glycogenolysis and glycolysis, with a resultant additional increase in ATP syntheses (Levy, 2006). With this increase in metabolism, there is a concomitant increase in reactive oxygen species (ROS) and reactive nitrogen species production in the tissue *via* a number of pathways (e.g. within the mitochondria, the xanthine oxidase pathway, NADP oxidase) (Powers *et al.*, 2011). In humans and animals, ROS also act as signalling molecules to aid in adaptation (e.g. increasing mitochondrial biogenesis) of muscle systems to be able to cope with increased contraction demand. Some of these adaptations include improved blood flow through capillarization, increased enzyme activities of the metabolic pathways and the upregulation of antioxidant pathways (French *et al.*, 2008; Powers *et al.*, 2016). Enzymes in the antioxidant pathways like superoxide dismutase require zinc, copper and manganese for optimal function. Superoxide dismutase converts superoxide to hydrogen peroxide, where after it is further neutralized to water by peroxiredoxins and glutathione peroxidase (requiring selenium as a co-factor), or reduced to water and oxygen (Cleveland and Kastan, 2000).

Fear and resultant flight during escape both cause an increase in muscle metabolism and therefore increased ROS production. Hence, if capture fear and escape–exertion are excessive, then overproduction of ROS may become a possibility (Barth *et al.*, 2007; Reardon and Allen, 2009). Excessive ROS are known to cause mitochondrial oxidative phosphorylation to uncouple, leading to heat production, which in turn may trigger cell death (Busiello *et al.*, 2015; Powers *et al.*, 2016). Thus, in theory, if the overproduction of ROS overwhelms the antioxidant defence system of an animal, it may lead to the build-up of highly reactive superoxide ions, which could be the cause of rhabdomyolysis, and the elevated body temperature observed in capture myopathy.

In the presence of iron molecules, superoxide may be converted to hydroxyl radicals, which is the strongest oxidant produced in biological systems and a potent trigger of cell death (Schrader and Fahimi, 2006; Augusto and Miyamoto, 2012). Large variations in iron content exist in skeletal muscles between species and, hence, may affect the rate of hydroxyl radical formation. Although not yet linked to capture myopathy, the higher iron concentration may be a cause or be a predisposing factor in certain species that are more susceptible to capture myopathy (Mostert and Hoffman, 2007). In support of this argument, iron supplementation in mice increased iron carriers (i.e. ferritin) by ~200%, and it also increased the activities of glutathione reductase and glutathione peroxidase by 30% and 220%, respectively. Exercise performance in these mice decreased substantially, and they were more prone to oxidative stress (Barth *et al.*, 2007). Additionally, when dietary iron is excessive, it causes copper deficiency, possibly by preventing copper absorption (Dashti *et al.*, 2015). Copper deficiency is known to result in decreased antioxidant (superoxide dismutase) activity in muscle tissues (Dashti *et al.*, 2015). With superoxide known to cause uncoupling in the mitochondria, leading to increased thermogenesis, continuous production of superoxide may be the cause of hyperthermia in capture myopathy and exertional heatstroke, even when muscle contraction has stopped for a prolonged duration Echtay *et al.* (2002).

#### Abnormal response of muscle metabolism to hormones may cause rhabdomyolysis

Past research has shown that hyperthermia and rhabdomyolysis may be caused by rapid surges in hormone levels. Specifically, higher-than-normal levels of thyroid hormone or noradrenalin have been shown to increase mitochondrial uncoupling (Mills *et al.*, 2004; Rusyniak and Sprague, 2006; Sprague *et al.*, 2007). Additionally, rhabdomyolysis has been induced when α_1_- and β_3_-adrenoreceptors were activated using various drugs that stimulate the sympathetic nervous system (Mills *et al.*, 2004; Rusyniak and Sprague, 2006). In theory, different responses to stress, the amount of hormone released, the sensitivity of skeletal muscles to these hormones and differences in mitochondrial uncoupling between species and individual animals may therefore explain why some animals are more susceptible to develop capture myopathy but requires further investigation.

#### Rhabdomyolysis can be caused by inherent muscle myopathies

Although metabolic myopathies are not believed to be the most common aetiology for exertional rhabdomyolysis in humans, they should be considered and eliminated as a possible cause (Rawson *et al.*, 2017). Similarly, they should be considered and investigated in cases of capture myopathy (Bartsch *et al.*, 1977; Capacchione and Muldoon, 2009). A number of metabolic myopathies identified in humans may cause muscle rhabdomyolysis (Guis *et al.*, 2005; van Adel and Tarnopolsky, 2009). These include genetic mutations in mitochondria, fatty acid oxidation and glycogen metabolism, resulting in an imbalance between energy supply and demand. Of these, carnitine palmitoyl transferase II and myophosphorylase deficiencies are well known to cause rhabdomyolysis (Guis *et al.*, 2005; Rawson *et al.*, 2017).

There is evidence that supports the involvement of metabolic myopathies in the various conditions that present with rhabdomyolysis. Wappler *et al.* (2001) found that 10 out of 12 patients who developed exertional heatstroke were tested positive for malignant hyperthermia. As mentioned before, this condition can be triggered in pigs by emotional stress, but whether this is the case in wild animals still needs to be determined (Antognini *et al.*, 1996; Aleman, 2008; Capacchione and Muldoon, 2009). PSSM in horses (and potentially in cetaceans) is a common cause of exertional rhabdomyolysis and therefore a highly plausible cause for capture myopathy (Valberg *et al.*, 1992; Aleman, 2008; Roe and Spraker, 2012; Sierra *et al.*, 2012). Thus, the presence of metabolic myopathies in wildlife should be investigated in more detail in susceptible species.

**Figure 1:**
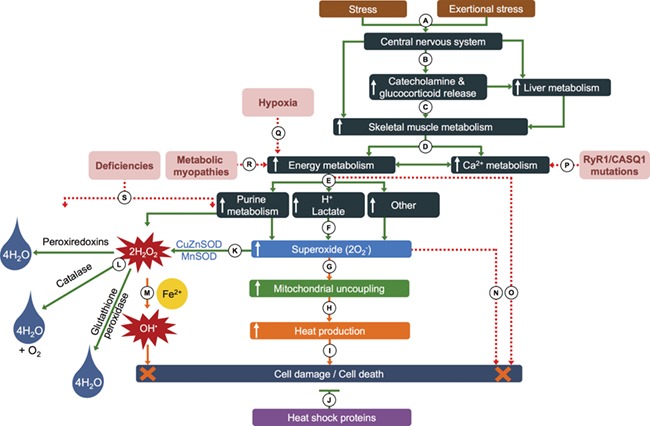
Simplified hypothesis for the possible pathomechanisms of capture myopathy and rhabdomyolysis in wild animals. (**A**) Stimuli in the form of fear and/or exertional stress (typical fight or flight response), with the central nervous system reacting to the stimuli. (**B**) Increase in sympathetic nervous activation and increased adrenalin, noradrenalin, dopamine and glucocorticoid secretion and release, as well as increased liver metabolism and skeletal muscle activity. (**C**) Increased catecholamine secretion upregulates skeletal muscle metabolism. (**D**) Increased ATP production from glycogen breakdown and phosphagen pathways in response to the demand from skeletal muscle contraction—myosin ATPase activity, active Ca^2+^-resorption into sarcoplasmic reticulum and the Na^+^K^+^ATPase pumps. (**E**) The increased demand for ATP replenishment results in elevated purine metabolism, increased lactate and H^+^ production and other pathways resulting in (**F**) increased generation of reactive oxygen species (ROS), such as superoxide (O_2_^–^). (**G**) The increase in O_2_^–^ results in greater uncoupling of oxidative phosphorylation and (**H**) increases heat production from the skeletal muscle. (**I**) An elevation in muscle temperature increases the risk of muscle fibre damage and necrosis (**J**) but is counteracted by the protective effect of heat shock proteins. (**K**) O_2_^–^ is converted to hydrogen peroxide (H_2_O_2_) by superoxide dismutase (SOD), which requires zinc, copper and manganese to function optimally. (**L**) Three pathways neutralize the H_2_O_2_ to water (peroxiredoxins and glutathione peroxidase that requires selenium to function optimally) and oxygen (catalase). (**M**) If not neutralized, H_2_O_2_ may be converted to hydroxyradical molecules (OH·) through the Fenton reaction (involving iron) that can cause severe cellular damage. (**N**) Excess ROS especially in the form of O_2_^–^ may cause cellular damage. (**O**) A lack of ATP replenishment as a result of excessive metabolism [e.g. glycogen depletion or (**Q**) hypoxia] prevents the myosin–actin cross-bridges to detach (form of rigor) and leads to damaged muscle fibres through mechanical stretch. Mutations in receptors involved in (**P**) Ca^2+^ regulation or (**R**) ATP production can result in muscle damage through the same mechanism proposed in (O). (**S**) Mineral deficiencies (co-factors) within the oxidative stress pathway enzymes can lead to diminished antioxidant capacities, leading to excess ROS that may injure cell membranes.

### External predisposition to capture myopathy

#### Nutritional factors associated with oxidative stress and rhabdomyolysis

Antioxidants play an important role in reducing the ROS produced from the increased metabolism during capture (Bagley *et al.*, 2007; Reardon and Allen, 2009). Many of the antioxidant pathway enzymes require cofactors in the form of minerals such as zinc, copper, selenium or manganese to function optimally (Powers *et al.*, 2011). Previous research on a similar condition, namely porcine stress syndrome, indicated that zinc supplementation, either in fodder or injected prior to stress, decreased the formation of pale soft exudative lesions typically found in the heart (Häggendal *et al.*, 1987). Similar results were obtained when animals were pre-treated with a combination of vitamin E and selenium (Liu *et al.*, 2018). Therefore, a deficiency in these mineral cofactors for optimal antioxidant functioning may prevent the neutralization of ROS and lead to excessive cell damage and excessive uncoupling of oxidative phosphorylation in mitochondria (Schrader and Fahimi, 2006).

Sadly, there is limited to no evidence that any supplementation has a protective effect against the development of rhabdomyolysis and capture myopathy, but it must be studied (Banerjee *et al.*, 2003; Powers *et al.*, 2004; Valberg, 2009). Anecdotal reports that vitamin E and selenium supplementation may prevent chronic exertional rhabdomyolysis in horses do exist but lack scientific backing from clinical trials (Beech, 1997). A greater understanding of ROS metabolism and the antioxidant status in healthy wild animals is needed, and further investigations are required to determine the role that ROS plays in capture myopathy.

#### Lack of adaptive physiological mechanisms to protect against rhabdomyolysis

A lack of exercise may play a role in the development of exertional heatstroke in humans and tying up in horses (Harthoorn, 1976; Rae *et al.*, 2008; Casa *et al.*, 2012). Poor fitness levels, which usually occur in wild animals kept in confined spaces (i.e. enclosures, paddocks and public exhibits), may predispose them to capture myopathy (Harthoorn, 1976; Smith, 2005; Rae *et al.*, 2008). However, although free-roaming wild animals are likely to be fitter than the above, their fitness level is unlikely adequate to endure the overexertion caused by a capture event. In fact, impala in a wild setting probably do not have a high level of fitness as they only run for less than 5% of the distance they normally travel in 1 day (La Grange *et al.*, 2010). Harthoorn (1979) developed methods that involved exercise training of wild animals before translocation, based on the assumption that training would aid in reducing capture-related deaths. This training may increase fitness and have the benefit of increasing habituation to capture procedures, thus reducing stress responses. However, whether fitness or habituation to stressful procedures plays a role in reducing capture myopathy has not yet been determined.

The proposed mechanism by which exercise training could protect against capture myopathy would be through the upregulation of antioxidant pathways. Production of ROS is a normal occurrence of physical exercise and the inherent antioxidant pathways provide sufficient means of neutralizing these free radicals (Powers *et al.*, 1999). However, when the intensity of exercise is severe, and coupled with stress and anxiety, the antioxidant pathways may be overwhelmed with subsequent oxidative damage (Banerjee *et al.*, 2003; Alleman *et al.*, 2015). Regular exercise training may be protective against these effects as it readily upregulates the amount and activity of antioxidant enzymes, thus effectively reducing ROS and increasing cellular protection against oxidative damage (Yamashita *et al.*, 1999; Banerjee *et al.*, 2003; French *et al.*, 2008).

Another cellular adaptation to exercise is increased levels of heat shock proteins. These proteins are crucial protectors of cellular components during periods of stress. Specifically, exertion (such as exercise) leads to hyperthermia, oxidative stress and altered fuel metabolism. It has been shown that both heat shock proteins and antioxidant enzyme expression levels can increase within 3 to 5 days after exposure to stressors induced by mild exercise intensities (Noble *et al.*, 2008). In the event of a subsequent exposure to stressors, these adaptations protect the cellular components and result in a higher survival rate of cells (Tupling *et al.*, 2008). Thus, the integrity and function of heat shock proteins in cellular protection during episodes of stress and capture in wild animals also needs to be investigated.

#### Pre-existing conditions

Pre-existing diseases, infections and severe verminoses cannot be excluded in predisposing animals to capture myopathy. Additionally, underlying kidney damage from drinking water with high salinity in certain habitats can contribute to the development of capture myopathy (La Grange *et al.*, 2010; Herráez *et al.*, 2013). Conversely, although marine mammals live in a ‘high salinity habitat’, they do not consume salt water as a norm and get most of their water requirements from their food or as a metabolic by product (Ortiz, 2001). Female animals in their final trimester of gestation may also be at greater risk of developing capture myopathy. All these different factors should be investigated further to evaluate the risk that each condition may contribute (La Grange *et al.*, 2010; Herráez *et al.*, 2013).

## Hypothesis of rhabdomyolysis in capture myopathy

An integrated hypothesis for the mechanisms that can possibly contribute to causing rhabdomyolysis in capture myopathy (and exertional heat stroke) is proposed in Figure 1.

Once an animal is in survival mode and fearing for its life (fight or flight), overcompensation of its physiological responses may be detrimental to its survival. Exertion increases several metabolic pathways, leading to an increase in metabolic (e.g. ROS) and physiological (e.g. hyperthermia) by-products that stress normal cellular functions. Usually, these stressors are neutralized to some extent by internal processes, such as the antioxidant system and increase blood flow to the periphery to dissipate heat or a feedback to the brain to stop exercising. Chronic and repetitive exposure to low levels of these stressors should lead to positive adaptations that may delay the onset and protect against the development of rhabdomyolysis. However, aggravating elements, such as genetic (e.g. metabolic myopathies) and environmental (e.g. lack of minerals in diets) factors, may predispose a stressed animal to fatal rhabdomyolysis. It therefore is essential that these hypothetical causes and proposed mechanisms be systematically investigated in wildlife models.

## Conclusion

(1) Capture myopathy is a condition that can kill many wildlife species; it is characterized by severe muscle rhabdomyolysis, kidney failure and elevated body temperatures. It presents with very similar symptoms and pathophysiology compared to human and domestic animal conditions with a rhabdomyolysis component.

(2) To date, no cure exists for capture myopathy.

(3) Good planning and available resources before a capture event are currently the best way in improving survival rates of wild animals.

(4) Due to a lack of scientific studies, various intrinsic and extrinsic factors that are known causes of muscle rhabdomyolysis in humans and animals have not yet been implicated as a cause for capture myopathy.

(5) There is an urgency to adequately determine the pathophysiology, triggers and predisposing factors that induce capture myopathy in order to identify targets for treatment, to ensure animal welfare and the survival of already endangered species.

### Conservation implications

Climate change, poaching and habitat loss increasingly threatens wildlife globally and is of increased concern for conservation in arid and semi-arid continents like Africa. The continent currently faces a tragic extinction epidemic of a multitude of species where translocation efforts may often be the only hope.

The need for increased success rates in the capture and translocation efforts of large mammals is thus of crucial importance and improvements can only be achieved by better understanding the causes and pathophysiology of capture myopathy.

## Dedication

We dedicate this review to the late Dr Antonie Marinus Harthoorn, whose pioneering work on capture myopathy has inspired our research endeavours.
